# Comparison of brain connectomes by MRI and genomics and its implication in Alzheimer’s disease

**DOI:** 10.1186/s12916-019-1488-1

**Published:** 2020-02-06

**Authors:** Young Jae Woo, Panos Roussos, Vahram Haroutunian, Pavel Katsel, Samuel Gandy, Eric E. Schadt, Jun Zhu, Alzheimer Disease Neuroimaging Initiative (ADNI)

**Affiliations:** 1 Department of Genetics and Genomic Sciences, Icahn School of Medicine at Mount Sinai, New York, NY 10029 USA; 2 Department of Psychiatry, Icahn School of Medicine at Mount Sinai, New York, NY 10029 USA; 3 Department of Neuroscience, Icahn School of Medicine at Mount Sinai, New York, NY 10029 USA; 4 Department of Neurology, Icahn School of Medicine at Mount Sinai, New York, NY 10029 USA; 5 Sema4, Stamford, CT 06902 USA

**Keywords:** Alzheimer’s disease, Diffusion tensor imaging, Transcriptome, Post-mortem brain, Imaging transcriptomics, Toll-like receptor signaling

## Abstract

**Background:**

The human brain is complex and interconnected structurally. Brain connectome change is associated with Alzheimer’s disease (AD) and other neurodegenerative diseases. Genetics and genomics studies have identified molecular changes in AD; however, the results are often limited to isolated brain regions and are difficult to interpret its findings in respect to brain connectome. The mechanisms of how one brain region impacts the molecular pathways in other regions have not been systematically studied. And how the brain regions susceptible to AD pathology interact with each other at the transcriptome level and how these interactions relate to brain connectome change are unclear.

**Methods:**

Here, we compared structural brain connectomes defined by probabilistic tracts using diffusion magnetic resonance imaging data in Alzheimer’s Disease Neuroimaging Initiative database and a brain transcriptome dataset covering 17 brain regions.

**Results:**

We observed that the changes in diffusion measures associated with AD diagnosis status and the associations were replicated in an independent cohort. The result suggests that disease associated white matter changes are focal. Analysis of the brain connectome by genomic data, tissue-tissue transcriptional synchronization between 17 brain regions, indicates that the regions connected by AD-associated tracts were likely connected at the transcriptome level with high number of tissue-to-tissue correlated (TTC) gene pairs (*P* = 0.03). And genes involved in TTC gene pairs between white matter tract connected brain regions were enriched in signaling pathways (*P* = 6.08 × 10^−9^). Further pathway interaction analysis identified ionotropic glutamate receptor pathway and Toll receptor signaling pathways to be important for tissue-tissue synchronization at the transcriptome level. Transcript profile entailing Toll receptor signaling in the blood was significantly associated with diffusion properties of white matter tracts, notable association between fractional anisotropy and bilateral cingulum angular bundles (*P*_permutation_ = 1.0 × 10^−2^ and 4.9 × 10^−4^ for left and right respectively).

**Conclusions:**

In summary, our study suggests that brain connectomes defined by MRI and transcriptome data overlap with each other.

## Background

Neural connections comprising a nervous system are often described in complicated anatomical networks. Much of the human brain connectome has been assessed using magnetic resonance imaging (MRI) where functional MRI and diffusion MRI (dMRI) can measure correlated neural activity and structural connectivity of the brain in vivo, respectively [1, 2]. Various neurological diseases such as Alzheimer’s disease (AD) are associated with disruption of the brain connectome and studies show that the course of AD continuum is associated with the changes in brain network architecture [3–5]. Although our knowledge regarding the connectome changes in AD is abundant, understanding the molecular consequences or causes of brain connectome changes is lacking.

Gene expression signatures carry important information for understanding structural and functional brain connectivity. It has been shown that the connectivity in rodent brains can be predicted from mouse brain expression data [6, 7]. Brain connectivity based on blood-oxygen-level-dependent signals at a resting state is significantly associated with correlations between gene expression of human brain segments [8]. However, the transcriptomic studies of AD are often limited to isolated brain regions such as the hippocampus or dorsolateral prefrontal cortex alone and are difficult to interpret its findings in respect to the brain connectome when its relation is not examined together [9, 10]. The mechanisms of how one brain region impacts molecular pathways in other regions, especially how the brain regions susceptible to AD pathology interact with each other at the transcriptome level, have not been systematically studied.

Here, we performed imaging-transcriptomic study analyses of brain connectomes based on dMRI imaging data from Alzheimer’s Disease Neuroimaging Initiative (ADNI) and a brain transcriptome dataset covering 17 brain regions [11–13]. Unlike traditional imaging genetic association analyses, where the goal is to identify the relationship between genetic variation and the changes in neurological traits [14, 15], the analyses here focused in spatial correlations between gene expression and structural brain connectivity. We hypothesize that different brain regions are synchronized at the molecular level (genomic connectome), partially facilitated by white matter tracts (structural connectome). Dysfunction of genomic connectome may associate with neurological diseases and reflect genetic propensity underlying AD etiology. To test our hypothesis, we (1) identified white matter tracts associated with AD based on dMRI and replicated them in an independent cohort [16], (2) identified brain regions connected by white matter tracts, (3) compared structural brain connections and genomic brain connections defined as tissue-to-tissue correlations (TTCs) at the transcription level, and (4) identified biological pathways involved in TTCs in structurally connected brain regions [17, 18].

## Methods

### Neuroimaging analysis

Data used in the preparation of this study were obtained from the ADNI database (http://adni.loni.usc.edu). The ADNI was launched in 2003 as a public-private partnership, led by Principal Investigator Michael W. Weiner, MD. The primary goal of ADNI has been to test whether serial magnetic resonance imaging (MRI), positron emission tomography (PET), other biological markers, and clinical and neuropsychological assessment can be combined to measure the progression of mild cognitive impairment (MCI) and early Alzheimer’s disease (AD). There were 232 ADNI2 subjects and 621 ADNI3 subjects with both T1 and diffusion-weighted MRI images at baseline when we downloaded the data on October 2018 from the Laboratory of Neuro-Imaging (http://adni.loni.usc.edu) [11]. All images were converted from DICOM to NIFTI using DCM2NII software (University of South Carolina, SC, USA) [19]. All anatomical regions of interests (ROIs) were segmented using FreeSurfer 6.0, 64-bit version (Massachusetts General Hospital, MA, USA) [20]. The FreeSurfer pipeline included motion correction of volumetric T1-weighted imaged, stereotaxic space transformation, intensity non-uniformity correction, removal of non-brain tissue, tessellation of gray/white matter boundaries via surface modeling, automatic topology correction, and surface deformation followed by intensity gradient that optimally defined tissue borders where the greatest shift in intensity defined the transition into the other tissue. Image outputs were visually checked for each subject. Segmentation of ROIs was conducted based on “Desikan-Killiany” cortical atlas [21]. Diffusion-weighted images were preprocessed using FSL 5.0.10 (Wellcome Center, Oxford, UK) [22]. Diffusion imaging pipeline included brain extraction, susceptibility-induced distortion correction, eddy current and motion correction, individuals’ axial diffusivity (AxD), radial diffusivity (RD), mean diffusivity (MD), and fractional anisotropy (FA) estimation, and diffusion uncertainty map calculation using BEDPOSTX [23–25]. Probabilistic tractography was performed using TRActs Constrained by UnderLying Anatomy (TRACULA), and 18 tracts were derived (Massachusetts General Hospital, MA, USA) [26]. The 18 tracts are forceps major, forceps minor, left anterior thalamic radiations (L-ATR), left cingulum—angular bundle (L-CAB), left cingulum—cingulate gyrus (L-CCG), left corticospinal tract (L-CST), left inferior longitudinal fasciculus (L-ILF), left superior longitudinal fasciculus parietal (L-SLFP), left superior longitudinal fasciculus temporal (L-SLFT), left uncinate fasciculus (L-UNC), right anterior thalamic radiations (R-ATR), right cingulum—angular bundle (R-CAB), right cingulum—cingulate gyrus (R-CCG), right corticospinal tract (R-CST), right inferior longitudinal fasciculus (R-ILF), right superior longitudinal fasciculus parietal (R-SLFP), right superior longitudinal fasciculus temporal (R-SLFT), and right uncinate fasciculus (R-UNC). For each tract, volume, average length, mean AxD, mean RD, mean MD, and mean FA were calculated.

Imaging data for ADNI2 and ADNI3 cohorts were processed under identical procedure. After all quality checks which include both systematic error checks and visual inspections, 593 out of 621 ADNI3 subjects and 220 out of 232 ADNI2 subjects successfully finished all imaging processes. Among 593 ADNI3 subjects, 550 subjects had qualifying clinical measures where age and gender were available and diagnosis record was within 60 days of scan date. Among 220 imaging processed subjects in ADNI2, 210 subjects had matching relevant phenotypes also within 60 days of scan date. Fifty-one subjects overlapped between ADNI3 and ADNI2 cohorts that had finished all the image processing and phenotype matched with scan date. Therefore, we omitted overlapping subjects from ADNI3 and analyzed 499 subjects for ADNI3 as discovery cohort and 210 ADNI2 subjects as the replication cohort. Association between AD diagnosis and diffusion measures of each tract (volume, average length, AxD, RD, MD, FA) was examined via linear model adjusted for age, gender, and total brain volume. Outliers that were 3.5 SD away from the mean were removed from the model, and all statistics were false discovery rate (FDR) adjusted for multiple comparisons. Effect sizes (*β*) of associations with AD (diffusion measure *y* = β*AD after adjusting covariates) was also calculated from the models.

### Reach probability calculation

The probability of tract reaching a Desikan-Killiany atlas defined grey matter ROIs at its white matter border was estimated. One voxel deep grey matter mask that is neighboring white matter was derived for all 82 ROIs and defined as ROI target masks. Path distribution for each tract was trimmed to include white matter and ROI target masks only. The probability of tract reaching ROI at its white matter boundary was calculated by dividing the number of paths passing through each voxel by the total path number in trimmed tract. Finally, the normalized probabilities within ROI target masks were averaged for all ROIs and this was repeated for each tract (Additional file 1: Figure S1A). These were defined as “reach probability” of tract connecting to grey matter ROIs (18 tracts towards 82 ROIs). The non-zero reach probability followed an extreme value distribution (Additional file 1: Figure S2A). We defined connections based on the empirical cumulative distribution function reflection point (reach probability = 0.002). At the cutoff, 203 of ROI-tract pairs were connected by a white matter tract. This effectively isolated some ROIs to specific tracts such as L-hippocampus was connected to L-CAB but not with L-SLFT or L-SLFP (Additional file 2: Table S1).

### Tissue-to-tissue correlated gene identification

Post-mortem brain tissues curated by Mount Sinai Hospital were analyzed where gene expression for 17 brain regions limited to the left hemisphere were available [12, 13]. The transcriptome data was made up of maximum 63 subjects, and any two brain regions were shared by 30–51 subjects (Additional file 2: Table S2). The methods and cohort characteristics for this dataset have been described in detail [12, 13]. The 17 brain regions were frontal pole (FP), occipital visual cortex (OVC), inferior temporal gyrus (ITG), middle temporal gyrus (MTG), superior temporal gyrus (STG), posterior cingulate cortex (PCC), anterior cingulate cortex (ACC), parahippocampal gyrus (PHG), temporal pole (TP), precentral gyrus (PCG), inferior frontal cortex (IFC), dorsolateral prefrontal cortex (DLPFC), superior parietal lobule (SPL), prefrontal cortex (PFC), caudate nucleus (CN), hippocampus (HIP), and putamen (PUT) (Additional file 2: Table S2). The gene expression values were adjusted for age, sex, post-mortem interval, pH, ethnicity, and Braak staging scores. The adjustment removed potential batch-driven gene-gene correlations, such as both genes were up in an ethnic group or disease diagnosis group, but enhanced gene-gene correlations that were consistent among ethnic groups or disease diagnosis groups, and etc. Spearman correlation was used in identifying TTC gene pairs between 136 pairs of brain regions (17 × 16/2). Significant TTC gene pairs were identified at genome-wide threshold *P*-value < 1 × 10^−8^ as defined in previous TTC study [17]. Significant TTC gene pairs were counted for 136 brain region pairs (Additional file 2: Table S3).

### Bipartite clustering

Significant TTC signals were discretized as binary values, and Barber’s modularity was maximized which identifies two-mode networks of disjoint gene sets such that interaction only occurs with genes of another brain region [27]. LPAb+ algorithm outperforms other methods for bipartite networks [28, 29], and we utilized its two-stage procedure where first “bottom-up” step propagates labels iteratively to maximize node-by-node modularity and second “top-down” step joins modules together to increase network modularity [30]. Different random initialization of node selection was performed five times for all 136 ROI pairs and confirmed that the maximized modularity converged to same optimal solution. For each ROI pair, bipartite modules with more than 1000 interactions (TTC gene pairs) were selected and genes within modules were pooled for each tissue before conducting pathway enrichment analysis.

### Pathway enrichment analysis

Curated pathways from Protein Analysis Through Evolutionary Relationships (PANTHER) database v.14.1 were analyzed [31]. Among 177 curated pathways available, eight pathways made up of drosophila-specific pathways were omitted (P06209, P06211, P06212, P06213, P06214, P06215, P06216, P06217). The Fisher exact test was performed to assess overrepresentation of our gene lists in each pathway, and all human genes (*n* = 20,996) were used as background. All pathway enrichment was corrected for FDR.

In order to infer broad biological insight from pathways overrepresented in the genes involved in TTC gene pairs, we created eight pathway categories that are biosynthesis, signaling, disease, physiology, development, gene regulation, metabolism, and catabolism for which detailed group identity for each pathway is listed in Additional file 2: Table S4. As post hoc analysis, after observing that a large number of associations were part of signaling pathway subgroup, we further divided signaling pathways into synaptic signaling, immune signaling, synaptic immune signaling, endocrine signaling, and unclassified (Additional file 2: Table S4). If the synthesized end product or degraded starting material served as a ligand in any synaptic, immune, or endocrine signaling, they were assigned as such (adrenaline and noradrenaline biosynthesis, aminobutyrate degradation, androgen/estrogen/progesterone biosynthesis, cobalamin biosynthesis, phenylethylamine degradation, vitamin B_6_ metabolism, gamma-aminobutyric acid synthesis, histamine synthesis, vasopressin synthesis, vitamin D metabolism and pathway, bupropion degradation, nicotine degradation). Oxidative stress response (P00046) was categorized as immune signaling.

### Pathway interaction analysis

For a ROI pair, molecular pathways significantly enriched in genes involved in TTC gene pairs were binarized for each ROIs (FDR < 0.05) (Fig. 4). Binarized pathway associations were matched between ROI1 and ROI2 that are paired in bipartite clustering step and were transformed into adjacency matrix (Additional file 1: Figure S3A). This defines pathway interaction between ROI pairs. There were three types of ROI pairs: (1) ROI pairs not connected by tracts (not-bound), (2) ROI pairs bound by tracts (tract-bound), and (3) ROI pairs bound by AD-associated tracts (AD-tract-bound). Among 136 ROI pairs, there were 72 not-bound, 64 tract-bound, and 43 AD-tract-bound. Proportion of pathway interactions in each group was calculated by normalizing the summed adjacency matrices by the number of ROI pairs (Additional file 1: Figure S3B). The chi-square test was performed comparing the proportion of pathway interactions for the tract-bound and AD-tract-bound groups. Both comparisons were compared against the not-bound group (Additional file 1: Table S5–6). The *P*-values were −log_10_ transformed and were hierarchically clustered using Ward’s method for further analysis.

### Blood expression analysis

The ADNI study collected whole blood samples for 811 subjects at baseline, which were processed using Qiagen PAXgene Blood RNA Kit (Germantown, MD, USA) [32]. Gene expression was profiled using Affymetrix Human Genome U219 Array (Affymetrix, Santa Clara, CA, USA) and was preprocessed using the Robust Multi-chip Average normalization method [33]. All quality check (QC) procedures were performed by ADNI Genetics Core which include RNA QC using Nanodrop and Agilent Bioanalyzer, overall array assay QC using Affymetrix Expression Console software and Partek Genomic Suite 6.6, sex verification, and sample identity prediction using Omni2.5 M genotype [32]. Quality-controlled transcriptome data was available for 744 subjects at 49,385 probe level and was downloaded from http://adni.loni.usc.edu. Blood transcriptome data was available for 102 ADNI2 subjects with successfully processed diffusion procedure and year at which PaxGene sample was collected matching with scan year. Toll receptor signaling pathway was represented by 49 genes spanning 129 probes in the transcriptome data [31], and probe-level expressions were collapsed to gene-level using mean-max method [34]. The association between diffusion measures for each tract and gene expression was examined according to the following linear model: *Diffusion measure*~*α* + *Gene Expression* + *Sex* + *Age* + *RIN* + (1| *AffyPlate*) + *ε* where *α* is intercept, *ε* is random error, and *RIN* is RNA integrity number. The aggregate effect of 49 Toll receptor signaling genes unto each diffusion measures was determined using sum of *χ*^2^ method [35, 36], and its significance was evaluated by 100,000 permutations (*P*_permutation_ < 0.05).

### Statistical analysis and visualizations

All statistical analyses were performed using Julia 1.0.3 (MIT, Cambridge, MA) [37]. The networks were visualized using spring-affinity algorithm. All heatmaps were drawn in R using Ward’s method for hierarchical clustering (R Core Team, Vienna, Austria) [38].

## Results

### Brain connectome by dMRI and associations with AD diagnosis

The ADNI3 cohort (*n* = 449, the “Methods” section) [11], consisting of 347 healthy controls, 118 mild cognitive impaired (MCI), and 34 AD patients, was interrogated for characterizing diffusion measures in 18 tracts derived using TRACULA [26] (Table 1). The brain volumes were positively correlated with MD and RD in all tracts (Additional file 1: Figure S4). RD was more significantly correlated with volume than MD. The average length of tracts was negatively correlated with MD and RD where MD was more correlated with tract average lengths than RD. In all 18 tracts of interest, AxD was positively correlated with FA and RD was positively correlated with MD.

**Table 1:** Demographic of ADNI2 and ADNI3

	Age (years)	Gender	Ethnicity						Diagnosis		
	Mean ± std	Males	Am. Indian/Alaskan	Asian	Black	White	More than one	Unknown	CN	MCI	Dementia
ADNI3 (*n* = 499)	73.8 ± 8.4	229 (45.9%)	1 (0.2%)	6 (1.2%)	15 (3.0%)	467 (93.6%)	8 (1.6%)	2 (0.4%)	347	118	34
ADNI2 (*n* = 210)	73.5 ± 6.8	120 (57.1%)	1 (0.5%)	6 (2.9%)	8 (3.8%)	192 (91.4%)	3 (1.4%)	0 (0.0%)	75	91	44

**Fig. 1:**
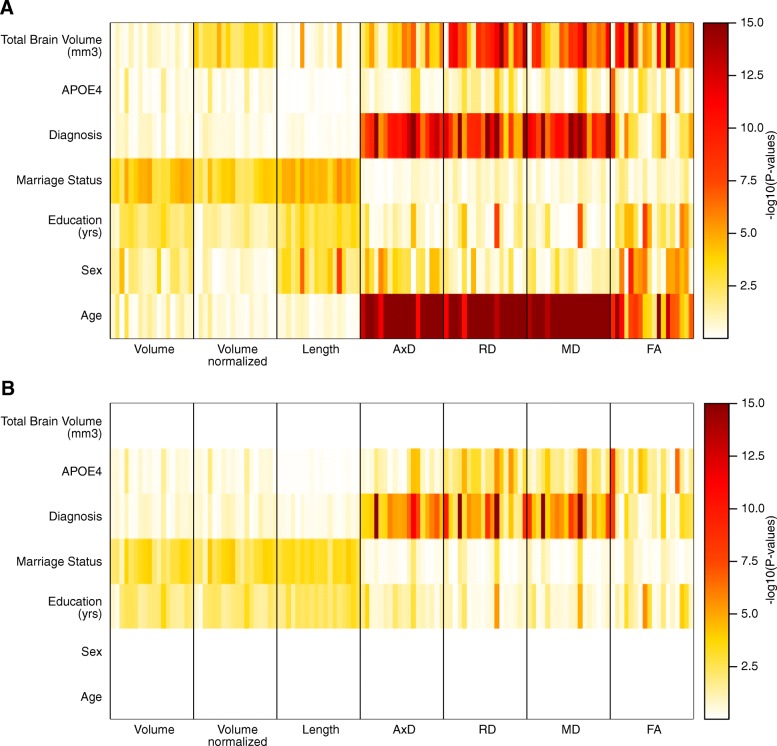
Association between diffusion properties of 18 major tracts and covariates. **a** For 18 major tracts, covariates age, sex, years of education, marriage status, clinical diagnosis, APOE4 genotype, and total brain volume were tested against tract measures that are volume, volume normalized by total brain volume, tract length, axial diffusivity (AxD), radial diffusivity (RD), mean diffusivity (MD), and fractional anisotropy (FA). Rows of the heatmaps represent covariates. Individual association was −log10 transformed and displayed as heatmaps. Columns are organized by tract measure types separated by black vertical lines. For each tract measure type, individual tract associations are arranged in the following orders from left to right: forceps major, forceps minor, left anterior thalamic radiations (L-ATR), left cingulum—angular bundle (L-CAB), left cingulum—cingulate gyrus (L-CCG), left corticospinal tract (L-CST), left inferior longitudinal fasciculus (L-ILF), left superior longitudinal fasciculus parietal (L-SLFP), left superior longitudinal fasciculus temporal (L-SLFT), left uncinate fasciculus (L-UNC), right anterior thalamic radiations (R-ATR), right cingulum—angular bundle (R-CAB), right cingulum—cingulate gyrus (R-CCG), right corticospinal tract (R-CST), right inferior longitudinal fasciculus (R-ILF), right superior longitudinal fasciculus parietal (R-SLFP), right superior longitudinal fasciculus temporal (R-SLFT), right uncinate fasciculus (R-UNC). **b** Tract measures were adjusted for age, sex, and total brain volume before association testing, and only diagnosis associations remain strongly associated with diffusion-related measures AxD, RD, MD, and FA

The diffusion measures were compared against age, sex, years of education, marriage status, APOE4 genotype, and total brain volume (TBV) and disease diagnosis (Fig. 1a). Age was significantly associated with AxD, RD, and MD in all tracts (*P*-values = 2.2 × 10^−5^ ~ 2.4 × 10^−17^) and with FA in a subset of tracts (16 out of 18 tracts below *P*-value < 0.05, *P*_min_ = 4.2 × 10^−12^), consistent with reports in literature [39]. TBV was associated with RD, MD, and FA in a number of tracts. Disease diagnosis status was associated with AxD, RD, and MD in a large number of tracts similar to findings in other studies [40]. After adjusting age, sex, and TBV effects, only disease diagnosis status remained significantly associated with the diffusion measures (Fig. 1b). Among all diffusion measures in 18 tracts, we identified 34 significant disease associations in a data-driven manner with AxD, RD, MD, and FA in a number of tracts at FDR < 0.05 (Table 2). There was no AD diagnosis status association with bilateral CST and FMajor which are responsible for motor and visual functions.

**Table 2:** Association between diffusion parameters and disease (FDR estimated independently)

	ADNI3 (n = 499)						ADNI2 (n = 210)					
Tract	Volume	Length	AxD	RD	MD	FA	Volume	Length	AxD	RD	MD	FA
FMajor	0.41	0.83	0.55	0.09	0.14	*0.02*	0.89	0.18	0.64	0.07	0.07	0.08
Fminor	0.41	0.85	0.06	*4.1 × 10*^*−3*^	*0.01*	0.41	0.44	0.59	0.37	0.26	0.22	0.16
L-ATR	0.73	0.86	*0.04*	0.16	0.08	0.90	0.30	0.81	0.63	0.54	0.52	0.65
L-CAB**	0.41	0.74	*2.1 × 10*^*−5*^	*1.9 × 10*^*−7*^	*3.0 × 10*^*−7*^	*0.04*	*0.01*	0.10	*2.7 × 10*^*−4*†^	*9.3 × 10*^*− 7*†^	*9.2 × 10*^*− 7*†^	*0.05*^†^
L-CCG	0.95	0.96	0.06	*0.02*	*0.01*	0.39	0.65	0.65	0.08	0.95	0.34	0.46
L-CST	0.86	0.83	0.21	0.21	0.17	0.40	0.62	0.08	0.06	0.90	0.51	0.32
L-ILF**	0.71	0.85	*0.02*	*0.04*	*0.02*	0.88	0.54	0.63	*0.04*^†^	0.19	0.05	0.65
L-SLFP**	0.41	0.88	*0.03*	0.09	*0.02*	0.88	0.59	0.90	*0.02*^†^	0.67	0.22	0.30
L-SLFT**	0.49	0.88	*0.03*	0.11	*0.04*	0.71	0.63	0.65	*1.2 × 10*^*−3*†^	0.90	0.18	0.08
L-UNC	0.85	0.85	*0.03*	*1.1 × 10*^*−3*^	*2.0 × 10*^*−3*^	0.55	0.08	0.63	0.18	0.11	0.08	0.76
R-ATR	0.90	0.87	*0.02*	0.10	0.05	0.75	*0.05*	0.65	0.72	0.46	0.51	0.63
R-CAB**	0.60	0.63	*4.8 × 10*^*−3*^	*1.2 × 10*^*−5*^	*2.3 × 10*^*−5*^	*0.04*	0.06	0.06	*1.2 × 10*^*−3*†^	*4.2 × 10*^*− 3*†^	*6.6 × 10*^*−4*†^	0.54
R-CCG	0.93	0.85	*4.8 × 10*^*−3*^	0.10	*0.01*	0.89	0.69	0.86	0.08	0.90	0.51	0.58
R-CST	0.90	0.95	0.08	0.26	0.11	0.49	0.58	0.24	0.32	0.90	0.69	0.54
R-ILF	0.34	0.83	*0.04*	0.07	*0.04*	0.95	0.95	0.95	0.09	0.86	0.46	0.95
R-SLFP	0.75	0.67	*0.02*	0.41	0.10	0.06	0.65	0.65	0.07	0.89	0.51	0.38
R-SLFT**	0.90	0.85	*0.01*	0.39	0.06	0.07	0.41	0.90	*0.05*^†^	0.65	0.65	0.06
R-UNC	0.75	0.85	*0.04*	*1.6 × 10*^*−3*^	*4.8 × 10*^*−3*^	0.20	0.13	0.79	0.54	0.86	0.65	0.76

The associations below FDR < 0.05 in each cohort study are presented in italics

**Tracts with associations identified in ADNI3 and replicated in ADNI2

^†^Associations identified in ADNI3 and replicated in ADNI2

**Fig. 2:**
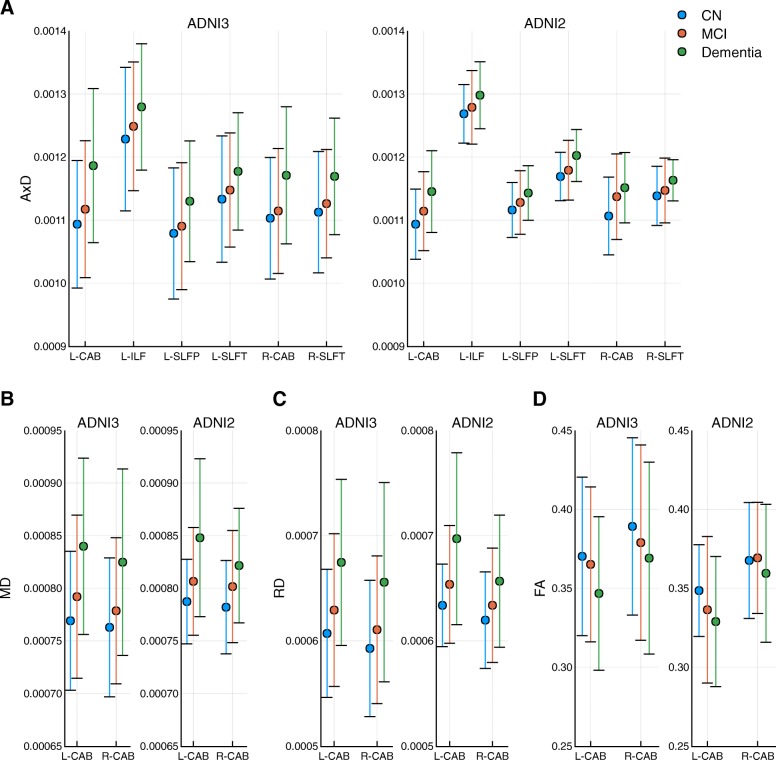
Eleven associations between diagnosis and diffusion parameters are replicated, and their change in direction is the same. **a** For AxD, L-CAB, L-ILF, L-SLFT, L-SLFP, R-CAB, and R-SLFT are significantly associated with diagnosis status where AxD increased with disease severity. This is observed in ADNI3 (*n* = 499) and is replicated in ADNI2 (*n* = 210). **b**, **c** For MD and RD, bilateral CABs are associated with diagnosis status and their change in direction was consistent in two independent cohorts. **d** FA in bilateral CABs are associated with diagnosis status in ADNI3 but only L-CAB association is replicated in ADNI2. The change in direction is consistent between two cohorts. AxD axial diffusivity, RD radial diffusivity, MD mean diffusivity, FA fractional anisotropy, L-CAB left cingulum—angular bundle, L-ILF left inferior longitudinal fasciculus, L-SLFP left superior longitudinal fasciculus parietal, L-SLFT left superior longitudinal fasciculus temporal, R-CAB right cingulum—angular bundle, R-SLFT right superior longitudinal fasciculus temporal

**Fig. 3:**
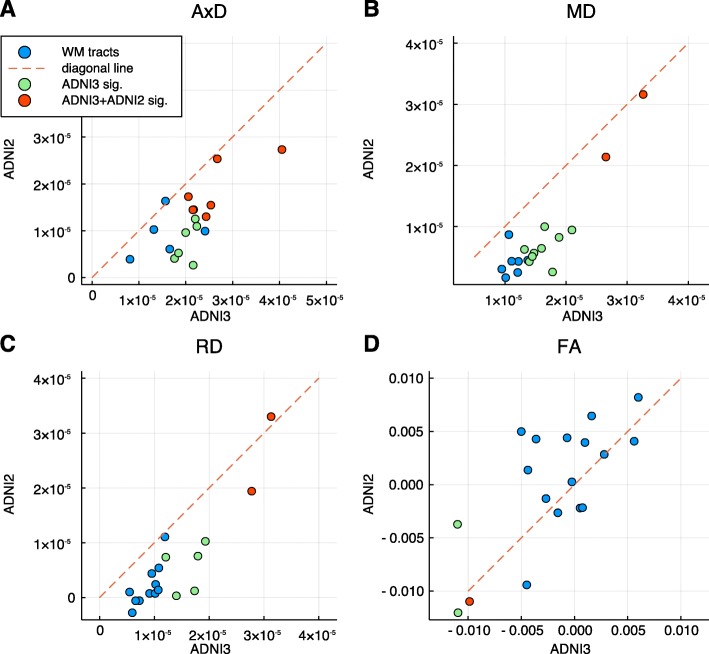
The replicated associations had larger effect sizes than non-replicated associations. The 2D scatter plot of effect sizes in ADNI3 and ADNI2 suggests concordance of associations in the two studies. Red circles are associations replicated in ADNI2, and light-green circles represent non-replicated associations. **a**–**c** The replicated associations in AxD, MD, and RD had larger effect sizes than non-replicated associations. **d** L-CAB association with FA had a large effect size relative to other comparisons and was replicated

The ADNI2 cohort (*n* = 210, the “Methods” section) [41], consisting of 75 healthy controls, 91 MCI, and 44 AD patients, is a cohort independent from the ADNI3 cohort (the “Methods” section). The same 18 tracts were derived using TRACULA [26]. The similar inter-relationships among diffusion measures, covariates, and disease diagnosis were observed (Additional file 2: Table S7). After adjusting age, sex, and TBV effects, 11 diffusion measures were significantly associated with disease diagnosis at FDR < 0.05 (Table 2, right) involving 4 of 18 tracts, bilateral CABs, bilateral SLFTs, L-SLFP, and L-ILF. Note that not only all the 11 associations overlapped with the 34 associations identified in ADNI3 cohort, but the direction of measure changes in response to diagnosis status was also replicated (Fig. 2). AxD, RD, and MD increased while FA decreased with disease diagnosis status (Fig. 2). The effect sizes and directions in ADNI2 and ADNI3 cohorts for the 34 associations identified in the ADNI3 cohort and the 11 replicated associations exhibited higher effect sizes than non-replicated associations (Fig. 3, Additional file 2: Table S8), suggesting a larger sample size is needed for replicating these associations of small effect sizes.

### Brain regions connected by different white matter tracts

We extracted path distribution information from each tract and calculated the probability of a tract reaching any Desikan-Killiany defined grey matter ROI [21]. Throughout this study, these measures were referred to as “reach probability.” Reach probability was developed to allow focusing on only major tracts and integrative analysis between neuroimaging and transcriptome datasets. The reach probability was limited to white matter boundary neighboring each respective ROI in order to avoid amplifying the connection to ROI based on within-ROI streamline propagations in the tractography processes. Reach probability was derived for 18 tracts towards 82 ROIs, and 607 out of 1476 (18 × 82) probabilities had zero reach probability (41%) (Additional file 2: Table S1). The non-zero reach probability followed an extreme value distribution (Additional file 1: Figure S2), and 203 ROI-tract pairs (33.4%) were identified (the “Methods” section).

### Brain connectome by tissue-tissue transcriptional synchronization

**Fig. 4:**
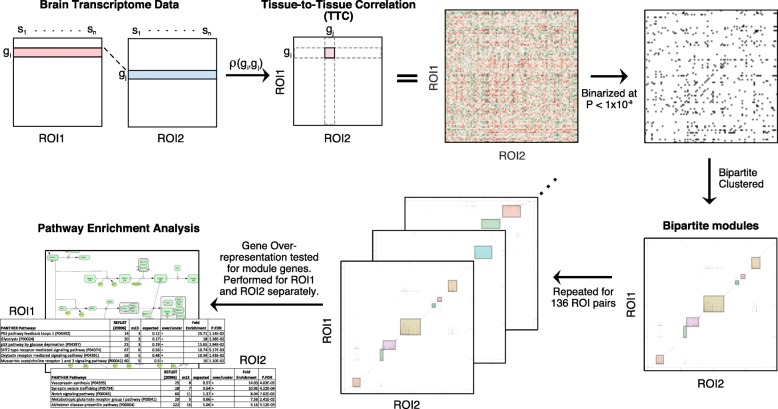
Schematic of brain region network analysis based on genetics. Tissue-to-tissue correlations (TTCs) are calculated between a pair of brain regions of interest (ROIs) for all gene combinations. Only the significant correlations are preserved (*P*-value < 1 × 10^−8^) [17] and are transformed into binary signals before bipartite clustering (see the “Methods” section for detail). This procedure is repeated for 136 possible brain region pairs (17 × 16/2). Only the bipartite modules with large number of clustered gene interactions (> 1000) are selected for each ROI producing 272 lists of genes (2 ROIs × 136). To examine how TTC genes are corroborating on shared molecular functions, pathway analysis is performed where only curated 169 pathways from PANTHER were examined. Overrepresented pathways were further analyzed (Figs. 6 and 7)

**Fig. 5:**
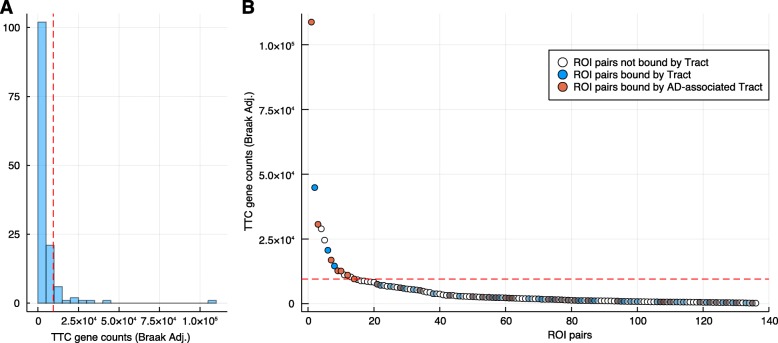
Total TTC gene counts for each ROI pairs examined. TTC genes were calculated after adjusting for age, sex, post-mortem interval, pH, ethnicity, and Braak staging scores. **a** Histogram of total TTC gene counts for all ROI pairs is displayed and top 10th percentile is demarcated by red dashed line. **b** ROI pairs are ranked by the total TTC gene counts and red dashed line represents top 10th percentile boundary. Clear circles are ROI pairs not bound by white matter tract, and all colored circles are ROI pairs bound by tracts where red circles are ROI pairs bound by AD-associated tracts and blue circles are ROI pairs bound by other tracts. All the ROI pairs below 10th percentile (red dashed line) are made partially transparent

In order to investigate molecular connections between brain regions, we analyzed tissue-tissue co-regulation [17, 18] of transcriptomic data covering 17 post-mortem brain regions (Fig. 4) [12, 13]. Subjects shared for each pair of brain regions were in the range of 30–51 subjects depending on post-mortem tissue availability. There were 136 (17 × 16/2) possible brain region pairs among 17 brain regions. Brain connectome is defined by TTC of all gene pairs after adjusting Braak score in order to examine consistent gene synchronization between brain regions with regard to different disease diagnosis groups. TTCs were adjusted for covariates such as age, sex, post-mortem interval, pH, and race [17] (detailed in the “Methods” section). The strength of brain region-region connections was measured by the number of significant TTC gene pairs. The distribution of significant TTC gene pair counts is shown in Fig. 5a for 136 region pairs, suggesting that only a fraction of brain regions were synchronized at the transcriptional level.

### Comparison of brain connectomes by diffusion MRI and genomics

The post-mortem brain regions were originally labeled according to the Brodmann area map [12] and were matched to brain regions in Desikan-Killiany cortical atlas [21] that was used in the above tractography analysis (Additional file 2: Table S2). Among 136 possible brain region pairs, 64 region pairs were connected by white matter tracts defined by tractography. Among 64 tract-bound region pairs, 28 pairs were connected by AD-associated tracts: L-CAB, L-ILF, L-SLFT, and L-SLFP. Among the top 10 percentile of region pairs (*n* = 14) containing the highest number of significant tissue-tissue correlated gene pairs, 10 were tract-bound (Fisher’s exact test, *P* = 0.057; Fig. 5b), and 7 out of top 10 percentile of region pairs (*n* = 14) were bound by AD-associated tracts (Fisher’s exact test, *P* = 0.03; Fig. 5b). This suggests that the brain connectomes defined by two different approaches overlap, especially for connections related to AD.

### Pathways associated with TTC gene pairs between different brain regions

**Fig. 6:**
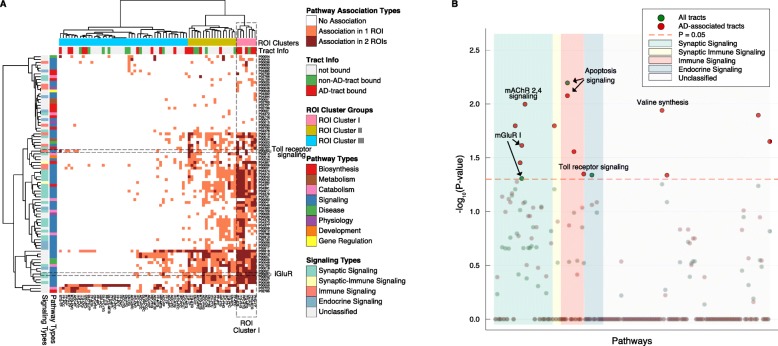
Pathways enriched by TTC genes. **a** Heatmap depicts all pathways overrepresented by TTC genes (FDR < 0.05). Only pathways and ROI pairs with significant associations are displayed. The rows are pathways, and columns are ROI pairs. In the heatmap, orange points to association between pathway and one ROI in its ROI pair (asymmetric gene synchronization), and brown is the association between pathway and both ROIs in its pair (symmetric gene synchronization). Two pathway categories, Pathway Types and Signaling Types are color labeled on the left (see the “Methods” section for details). ROI pairs are labeled with Tract Info and ROI clusters. ROI clusters is based on hierarchical clustering. **b** The association of TTC-associated pathways depending on ROI connections to white matter tracts was examined (see the “Methods” section for details). Each circle represents pathways where green circles are tract-dependent and red circles are AD-associated tract-dependent. Associations are −log10 transformed (*Y*-axis), and 169 pathways are organized by Signaling Types in the *X*-axis (different background color in the scatter plot). The pathways associated in both tract-dependent and AD-associated tract-dependent manner are indicated by arrows with pathway names. Orange dashed line delineates *P*-value = 0.05, and circles below this nominal significance are made partially transparent

Different brain regions were connected by white matter tracts and synchronized at the transcriptional level as shown above. To investigate whether any biological pathways were transcriptionally synchronized between brain region pairs, we constructed bipartite clusters of TTC gene pairs for all 136 ROI pairs and identified gene modules for each brain region in ROI pairs (Fig. 4). Genes in the modules were annotated using PANTHER database [31], and pathways enriched among these genes at FDR < 0.05 are listed in Additional file 2: Table S9. Among 169 pathways × 136 ROI pairs (22,984), 736 (3.2%) pathways to ROI pair associations were significant, covering 83 pathways and 69 ROI pairs (Fig. 6a). A large fraction (51/83 = 61.4%) of enriched pathways belonged to signaling pathways (Fisher exact test, *P* = 6.08 × 10^−9^, Fig. 6a). The 69 ROI pairs were clustered to 3 clusters according to enriched pathways (Fig. 6a). The ROI pairs in cluster I (Fig. 6a), which were connected by multiple pathways, were enriched for tract-bound ROI pairs and AD-associated tract-bound pairs (*P* = 0.04 and 0.01, respectively). This suggests that white matter tracts may serve as a mechanism of gene synchronization for signaling pathways, at least in brain regions present in ROI cluster I (PHG-TP, PHG-STG, ITG-MTG, ITG-PHG, IFC-ITG, PFC-STG, IFC-ITG).

Next, we examined whether any pathways were preferentially involved in TTCs of tract-bound or AD-associated tract-bound ROI pairs (Fig. 6b, detailed in the “Methods” section). Apoptosis signaling pathway (*P* = 0.006), EGF receptor signaling (*P* = 0.046), and metabotropic glutamate receptor (mGluR) I pathways (*P* = 0.049) were overrepresented in tract-bound region pairs (Additional file 2: Table S9). More pathways were preferentially involved in TTCs of AD-associated tract-bound ROI pairs, including apoptosis signaling (*P* = 0.008), muscarinic acetylcholine receptor (mAChR) 2 and 4 signaling pathway (*P* = 0.01), valine synthesis (*P* = 0.012), PI3 kinase pathway (*P* = 0.013), endothelin signaling pathway (*P* = 0.016), histamine synthesis (*P* = 0.016), p38 MAPK pathway (*P* = 0.02), mGluR I pathway (*P* = 0.024), inflammation mediated by chemokine and cytokine signaling pathway (*P* = 0.028), mGluR II pathway (*P* = 0.035), toll receptor signaling pathway (*P* = 0.045), adenine and hypoxanthine salvage pathway (*P* = 0.046) (Additional file 2: Table S9). The results suggest that signaling pathways, especially synaptic signaling and immune signaling pathways, involve in transcriptional synchronization between brain regions connected by white matter tracts.

### Toll receptor signaling pathway is overrepresented in both tract-bound and AD-associated tract-bound ROI pairs

**Fig. 7:**
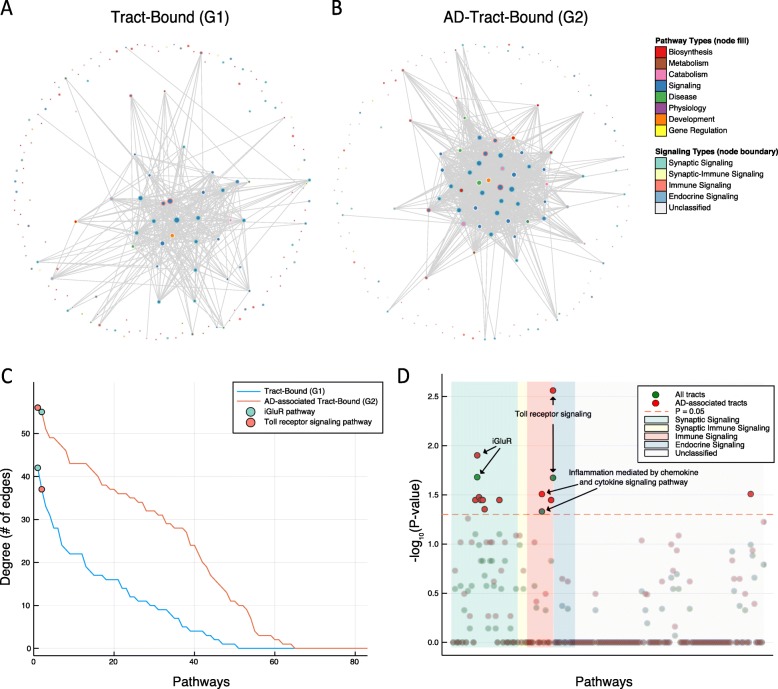
Ionotropic glutamate receptor (iGluR) and Toll receptor signaling pathways play key role in TTC genes across white matter tracts. Pathway interactions unique to ROI pairs depending on tract connections were examined using chi-square tests. The pathway interactions with nominal significance (*P* < 0.05) are illustrated as network edges and were drawn for **a** tract-bound (G1) and **b** AD-tract-bound (G2). The nodes are 169 pathways, and they are differentially colored by Pathway Types. Node boundary is color labeled by Signaling Types. The graph was constructed by Spring-Affinity algorithm. **c** The nodal degrees in both graphs G1 (blue) and G2 (red) are ranked for 83 pathways notated in Fig. 6a. Degree for iGluR pathway (cyan circle) and Toll receptor signaling pathway (orange circle) is overlaid in the plot. **d** Fisher exact test results for symmetric gene synchronization of pathways across tract-bound ROI pairs (green circles) and AD-tract-bound ROI pairs (red circles) are visualized. The circles are molecular pathways and are organized by Signaling Types in the *X*-axis. The *Y*-axis shows *P*-values that are negative log_10_ transformed. The TTC genes synchronized at the pathway level in both tract-bound and AD-associated tract-bound ROI pairs are indicated by arrows with pathway names. Orange dashed line delineates *P*-value = 0.05, and circles below this nominal significance were made partially transparent

Gene modules derived from bipartite clustering TTC gene pairs were enriched in specific molecular pathways, mostly related to signaling. However, biological pathways in one region may not reciprocally synchronize the same pathway in another brain region because each region is accountable for their own distinct roles. For instance, our analyses show that CN and ACC were structurally connected (Additional file 2: Table S1) and literature supports that they are functionally connected [42]. However, CN and ACC are enriched in different neuron types (dopaminergic [43] and spindle neurons [44], respectively) and are responsible for different biological processes that may be mediated by differing molecular functions. Using 169 curated pathways as generalizable domains of molecular functions [31], we investigate how pathways are differentially interacting between brain regions. We defined pathway interactions specific to tract-bound ROI pairs based on the chi-square test (*P* < 0.05, Additional file 2: Table S5) and the same analysis was performed for AD-associated tract-bound ROI pairs (Additional file 2: Table S6). Both pathway networks of tract-bound ROI pairs (G1) and AD-associated tract-bound ROI pairs (G2) were made up of nodes that are signaling related (Fig. 7a, b). G2 had a larger number of pathway interactions than G1 (Fig. 7c), and the node with the most number of edges in G2 was toll receptor signaling pathway (Additional file 2: Table S10). The top two nodes with the highest number of edges in the G1 were ionotropic glutamate receptor (iGluR) pathway and toll receptor signaling pathway (Fig. 7c).

Alternatively, TTC genes in ROI pairs may be involved in shared molecular functions (symmetric synchronization). We examined pathways associated in both brain regions in a pair (Additional file 2: Table S11). For genes in TTC gene pairs of tract-bound ROI pairs, iGluR pathway (*P* = 0.021), toll receptor signaling pathway (*P* = 0.021), inflammation mediated by chemokine and cytokine signaling pathway (*P* = 0.047) were significantly overrepresented (Fig. 7d). For genes in TTC gene pairs of AD-associated tract-bound region pairs, toll receptor signaling pathway (*P* = 0.003), iGluR pathway (*P* = 0.013), inflammation mediated by chemokine and cytokine signaling pathway (*P* = 0.031), PI3 kinase pathway (*P* = 0.031), mGluR group III pathway (*P* = 0.033), endothelin signaling pathway (*P* = 0.036), mGluR group II pathway (*P* = 0.036), mGluR group I pathway (*P* = 0.036), T cell activation (*P* = 0.036), 훽3 adrenergic receptor signaling pathway (*P* = 0.036), and mAChR 1 and 3 signaling pathway (*P* = 0.044) were preferentially involved (Fig. 7d).

### Toll receptor signaling genes in the blood associate with tract-wise diffusion measures in the brain

**Fig. 8:**
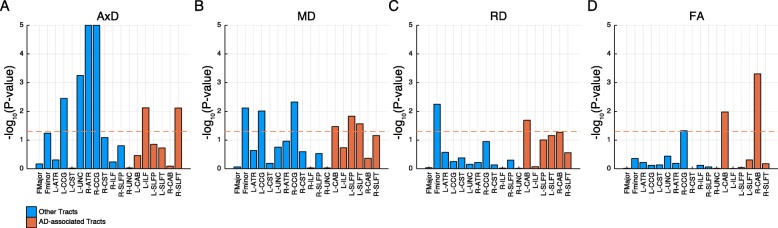
Toll receptor signaling-related gene expression in the blood associate with diffusion properties in the white matter. Gene expression in the blood and dMRI scans in shared subjects were analyzed (*n* = 102). The transcriptome effects of toll receptor signaling pathway on **a** AxD, **b** MD, **c** RD, and **d** FA in the 18 tracts were aggregated, and its *en masse* effect was approximated using 100,000x permutation. In all bar plots, AD-associated tracts replicated in two cohorts are in red, and other tracts are in blue. Orange dashed line represents *P*-value = 0.05 threshold for significance

Immune activities in the blood may reflect molecular states in the brain [45]. Because toll receptor signaling pathway was the most enriched pathway involved in symmetric synchronization between AD-associated tract-bound ROI pairs (Fig. 7d), we interrogated how toll receptor signaling-related genes’ expression in the blood associated with diffusion measures in the brain. There were 102 subjects with both blood expression data and dMRI scans in ADNI2 (the “Methods” section). We examined the pooled effect of 49 genes representing toll receptor signaling pathway [31] on diffusion measures of 18 tracts using sum of the chi-square method and compared them with the inferences based on 100,000 permutations [35, 36]. Multiple diffusion measures including AxD of R-ATR (*P* = 1.0 × 10^−5^), R-CCG (*P* = 1.0 × 10^−5^), L-UNC (*P* = 5.6 × 10^−4^), L-CCG (*P* = 3.5 × 10^−3^), L-ILF (*P* = 7.5 × 10^−3^), and R-SLFT (*P* = 7.6 × 10^−3^) were significantly associated with expression of genes in the toll receptor signaling pathway in the blood (Fig. 8a, Additional file 2: Table S12). RD (which measures diffusivity orthogonal to AxD) of forceps minor (*P* = 5.6 × 10^−3^) and L-CAB (*P* = 2.0 × 10^−2^) was significantly associated with the expression of toll receptor signaling-related genes in the blood (Fig. 8c). MD which captures diffusivity in all directions was associated in forceps minor (*P* = 7.6 × 10^−3^), bilateral CCGs (*P* = 9.7 × 10^−3^ and 4.7 × 10^−3^ left and right respectively), L-CAB (*P* = 3.4 × 10^−2^), L-SLFP (*P* = 1.5 × 10^−5^), and L-SLFT (*P* = 2.7 × 10^−2^) (Fig. 8b). FA which describes white matter integrity was associated in bilateral CABs (*P* = 1.0 × 10^−2^ and 4.9 × 10^−4^ left and right respectively) and R-CCG (*P* = 4.7 × 10^−2^) (Fig. 8d). Similar to diagnosis associations (Table 2), we did not observe any toll receptor signaling gene expression association with bilateral CST and forceps major which are responsible for motor and visual functions.

## Discussion

Tissues, organs and cell groups within organs, communicate with one another to perform biological functions in concert, and gene transcriptions are synchronized between tissues reflecting cross-tissue and cross-cell-group communications [17, 18]. In the brain, white matter tracts serve as an important medium of brain regional cross-talk [46–48], and we observed that large numbers of genes were synchronized at the transcriptional level in tract-bound brain regions (Fig. 5b). Gene modules derived from bipartite clustering of TTC gene pairs between tract-bound brain regions were significantly over-represented in signaling pathways (Fig. 6). Since axon bundles with synaptic connections constitute white matter tracts, identifying associations between mAChR, mGluR, and iGluR signaling pathways and TTC gene pairs were within our expectations (Fig. 6b). Toll receptor signaling pathway was the most enriched pathway in the symmetric gene synchronization between AD-associated tract-bound brain regions (Fig. 7). There are at least two potential mechanisms: (1) Toll-like receptor (TLR) signaling plays a role in brain region-to-region communication via white matter tract and (2) TLR signaling pathways in brain regions and in the blood are synchronized [49]. The association between diffusion measures in major tracts and toll receptor signaling pathway activity in blood convolutes the two potential mechanisms. Although the mechanism is not clear, our results suggest the immune system’s involvement in AD-associated brain region-to-region cross-talk.

TLRs play important roles in innate immunity in humans, and TLR activation in microglia due to neuropeptide aggregation is well established [50, 51]. However, the expression of TLRs is not limited to microglia [52, 53], but is also present in astrocytes [54], oligodendrocytes [55], neural progenitor cells [56, 57], and neurons [58]. The biology of TLRs is complex and goes beyond just recognizing pathogen-associated molecular patterns [59]. TLR3 can recognize double-stranded RNA for its activation [60], and the signaling cascade of TLRs varies for different neuronal cell types [61]. TLR2 and TLR4 are known to regulate hippocampal adult neurogenesis and neural progenitor cell differentiation [62]. TLR3 is associated with increased mature neurons in the hippocampus and enlarged dentate gyrus and the CA1 region [56]. TLR3 and TLR8 are present in the axonal tracts during the brain development and regulate neurite outgrowth and apoptosis [63–65]. In addition, differential expression of TLRs in human post-mortem brains are associated with alcohol addiction [66], depression [67, 68], and schizophrenia [69], and these neurological disorders are also associated with white matter abnormalities [70–72]. However, it is not known how TLRs may act on axonal degeneration and cross-communication between brain regions via axon fibers*.*

Diffusion-weighted imaging is a powerful tool in assessing microstructural changes of white matter in vivo, and diffusion parameters can capture white matter integrity [1]. In our work, TLR signaling expressions were associated with FA in bilateral CABs (Fig. 8). Because CABs have a strong connection to the hippocampus, white matter integrity measured by FA may be regulated by TLR signaling in the hippocampus and TLR-dependent adult neurogenesis [62]. AxD estimates parallel diffusivity along the direction of the highest diffusion and was significantly associated with expression of TLR signaling for bilateral-CCG, L-UNC, R-ATR, L-ILF, and R-SLFT. This suggests that TLR signaling may be involved in the loss of barriers restricting water diffusion in the associated tracts such as myelination level reduction or axon losses [73–75]. Although the association between diagnosis and diffusion measures in L-ILF and R-SLFT was replicated in the ADNI2 cohort, L-UNC, R-ATR, and R-CCG findings failed to replicate in the ADNI2 cohort (Table 2). L-CCG was only nominally significant (FDR < 0.1) in both ADNI3 and ADNI2 cohorts (Table 2). This suggests that expression variation of genes in the TLR signaling pathway might be more powerful in detecting microscopic white matter abnormalities in comparison to diagnosis status, and further study may allow developing blood biomarkers relevant to disease-associated white matter changes in vivo.

The sample size of ADNI3 was larger than the size of ADNI2 so that the ADNI3 study had a higher power to identify AD associations in diffusion imaging and not all associations were expected to be significant in the ADNI2. Besides the sample size, there were technical differences between the two cohorts [39, 76]. ADNI2 data was collected using older MR pulse sequence and was captured at 2.7-mm^3^ resolution. ADNI3 adopted the optimized protocol established by Human Connectome Project as the standard across multiple centers and gained higher resolution at 2.0 mm^3^ [11]. There were 16 and 50 research sites involved in ADNI2 and ADNI3 studies, respectively. Four hundred nine out of 499 images in the ADNI3 dataset were acquired from 37 research sites that were not included in the ADNI2 (Additional file 2: Table S13). The results from the multi-center studies are unlikely due to biases from a few sites. As noted in the “Methods” section, we included only imaging data of participants that were unique to ADNI3 as the ADNI3 cohort so that there was no overlap between the ADNI2 and ADNI3 cohorts in our analyses. The identified imaging-based disease associations were also consistent with known findings [77, 78]. All these results together suggest that the associations between neuroimaging features and AD are robust to the differences between ADNI3 and ADNI2. Additionally, the replicated associations had larger effect size than the non-replicated ones, suggesting associations of smaller effect sizes require a larger sample size to validate.

There are limitations in our analyses and ADNI studies in general. Majority of the participants in the ADNI2 and ADNI3 studies were white (91.4% and 93.6%, respectively). Even though some common associations between neuroimaging features and AD were identified in ADNI2 and ADNI3 cohorts, whether the associations hold in other ethnic groups needs further studies. Additionally, there were only 17 brain regions available to construct transcriptome-based brain connectome. The limited spatial resolution of this work may increase false negatives. The Allen Human Brain Atlas has more complete coverage of the brain spatially [79], but is limited to only 6 individuals whereas we conducted our study using 30–51 subjects depending on the brain region. Although spatially limited, our work is much better powered than the Allen Human Brain Atlas in examining correlated expression between brain regions and should better reflect the population information. Another limitation is that our study only examined gene synchronization by major white matter tracts whereas gene synchronization between two brain regions may be mediated through multiple mechanisms, including (1) direct neighbor (cis), (2) WM connected (trans), and (3) functionally connected (multi). Future works are needed to address these different gene synchronization models.

## Conclusion

Overall, this is the first study that investigates brain connectomes of white matter tracts and gene synchronization in human brains. For this, we developed a method that directly examines the enrichment of TTC genes in tract-bound brain regions and further performed molecular network analysis based on tract-wise connection information. Despite various limitations, we report that TTCs of genes in signaling pathways were significantly associated with brain regional cross-talk through white matter tracts. We further report that iGluR and toll receptor signaling pathways play a pivotal role in region-to-region communication and synaptic and immune interplay between brain regions may posit novel insights towards AD etiology.

## Supplementary information

**Additional file 1:** Supplementary figures.

**Additional file 2: Table S1.** Probability of tract reaching brain region (Reach probability). **Table S2.** Brain region mapping between Desikan-Killany atlas and post-mortem brain labels. **Table S3.** Number of Tissue-to-Tissue (TTC) correlated genes for each pair of Region-of-interests (ROIs). **Table S4.** Pathway list with annotated subtypes. **Table S5.** Pathway interactions in brain region pairs that are that are significantly different in tract-bound. **Table S6.** Pathway interactions in brain region pairs that are that are significantly different in AD-tract-bound. **Table S7.** Association between covariates and diffusion measures in each tract. **Table S8.** Effect sizes for associations in ADNI3 and ADNI2. **Table S9.** Pathway over-representation analysis between brain region pairs connected by white matter tracts and region pairs not connected by tracts. **Table S10.** Pathway interaction graph (degree). **Table S11.** Pathway over-representation analysis of symmetric gene synchronization in brain region pairs connected by white matter tracts. **Table S12.** Association between gene expression of Toll receptor signaling in the blood and diffusion measures in the brain. **Table S13.** Number of subjects in each study per site.

## Data Availability

ADNI is available at http://adni.loni.usc.edu. Post-mortem transcriptome data is available at GSE84422.

## Abbreviations

ACC: Anterior cingulate cortex
AD: Alzheimer’s disease
ADNI: Alzheimer’s Disease Neuroimaging Initiative
AxD: Axial diffusivity
CN: Caudate nucleus
DLPFC: Dorsolateral prefrontal cortex
dMRI: Diffusion MRI
FA: Fractional anisotropy
FDR: False discovery rate
FMajor: Forceps major
Fminor: Forceps minor
FP: Frontal pole
HIP: Hippocampus
IFC: Inferior frontal cortex
iGluR: Ionotropic glutamate receptor
ITG: Inferior temporal gyrus
L-ATR: Left anterior thalamic
L-CAB: Left cingulum—angular bundle
L-CCG: Left cingulum—cingulate gyrus
L-CST: Left corticospinal tract
L-ILF: Left inferior longitudinal fasciculus
L-SLFP: Left superior longitudinal fasciculus parietal
L-SLFT: Left superior longitudinal fasciculus temporal
L-UNC: Left uncinate fasciculus
mAChR: Muscarinic acetylcholine receptor
MCI: Mild cognitive impaired
MD: Mean diffusivity
mGluR: Metabotropic glutamate receptor
MRI: Magnetic resonance imaging
MTG: Middle temporal gyrus
OVC: Occipital visual cortex
PANTHER: Protein Analysis Through Evolutionary Relationships
PCC: Posterior cingulate cortex
PCG: Precentral gyrus
PFC: Prefrontal cortex
PHG: Parahippocampal gyrus
PUT: Putamen
QC: Quality check
R-ATR: Right anterior thalamic radiations
R-CAB: Right cingulum—angular bundle
R-CCG: Right cingulum—cingulate gyrus
R-CST: Right corticospinal tract
RD: Radial diffusivity
R-ILF: Right inferior longitudinal fasciculus
ROI: Region of interest
R-SLFP: Right superior longitudinal fasciculus parietal
R-SLFT: Right superior longitudinal fasciculus temporal
R-UNC: Right uncinate fasciculus
SPL: Superior parietal lobule
STG: Superior temporal gyrus
TBV: Total brain volume
TLRs: Toll-like receptors
TP: Temporal pole
TRACULA: TRActs Constrained by UnderLying Anatomy
TTCs: Tissue-to-tissue correlations
