# Supplementary material for: Comparison of brain connectomes by MRI and genomics and its implication in Alzheimer’s disease
Source: BMC Med. 2020 Feb 6;18:23. doi: 10.1186/s12916-019-1488-1 (PMC7003435; doi:10.1186/s12916-019-1488-1)
Supplement: Supplementary file 1 — Additional file 1: Supplementary figures. [file 12916_2019_1488_MOESM1_ESM.pdf]

Additional File 1

Additional File 1: Figure S1. Probability of tract reaching brain regions. Reach probability of tract connecting to 82 ROIs are visualized in **A)** heatmap and **B)** barplots for AD-associated tracts that have been replicated in two independent cohorts in the current study.

Additional File 1: Figure S2. Reach probability distribution. The distribution of reach probability is extremely heavy tailed and large proportion of reach probability measures are low. **A)** Histogram demonstrates this extreme value distribution. The orange dashed vertical line is reach probability of 0.002 and it cuts the distribution. This threshold is a reflection point based on Empirical Cumulative Distribution Function of reach probabilities. B) Empirical Cumulative Distribution Function of reaching probability below 5% is plotted. The orange dashed vertical line is reach probability of 0.002 and is an approximal reflection point.

Additional File 1: Figure S3. Pathway interaction analysis and results. Interaction between TTC genes by brain ROIs at the molecular pathways were examined. **A)** For a pair of ROIs, pathways significantly over-represented by TTC genes were binarized for each ROIs. The two binarized vectors for a ROI pair was transformed to adjacency matrix and this defines pathway interaction between a ROI pair. This was repeated for all 136 ROI pairs. **B)** The 136 adjacency matrices were grouped by white matter connection definition where not-tract-bound (72 ROI pairs), tract-bound (64 ROI pairs), and AD-tract-bound (34 ROI pairs). The heatmaps display proportion of pathway interactions normalized by number of ROI pairs in each group for not-tract-bound (left), tract-bound (middle), and AD-tract-bound (right). **C)** Significant difference in the proportion of pathway interaction between not-tract-bound and tract-bound was assessed using Chi-square test. The P-values were $-\log_{10}$ transformed and are hierarchically clustered. Strongly interacting pathways are highlighted in dashed rectangles. Pathway labels at two levels (Pathway Types and Signaling Types) are also added on the heatmap. **D)** Significant difference in the proportion of pathway interaction between not-tract-bound and AD-tract-bound was assessed using Chi-square test. Same heatmap display options as C) is applied here.

Additional File 1: Figure S4. Correlation between tract-specific measures in ADNI3. The correlation between Volume, average tract length (Length), Axial Diffusivity (AxD), Radial Diffusivity (RD), Mean Diffusivity (MD), and Fractional Anisotropy (FA) were examined for each tract.

Figure S1

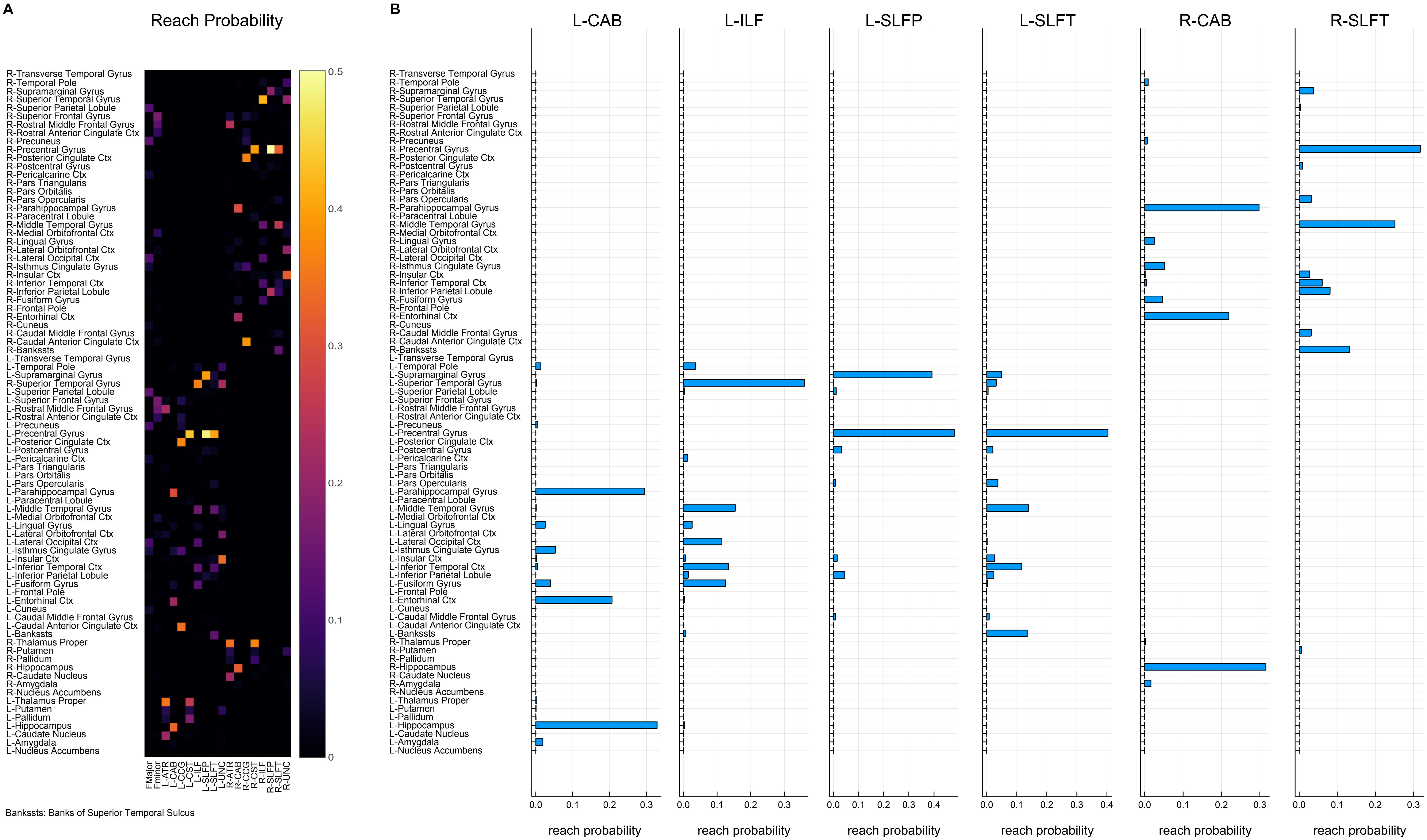

Bankssts: Banks of Superior Temporal Sulcus

Figure S2

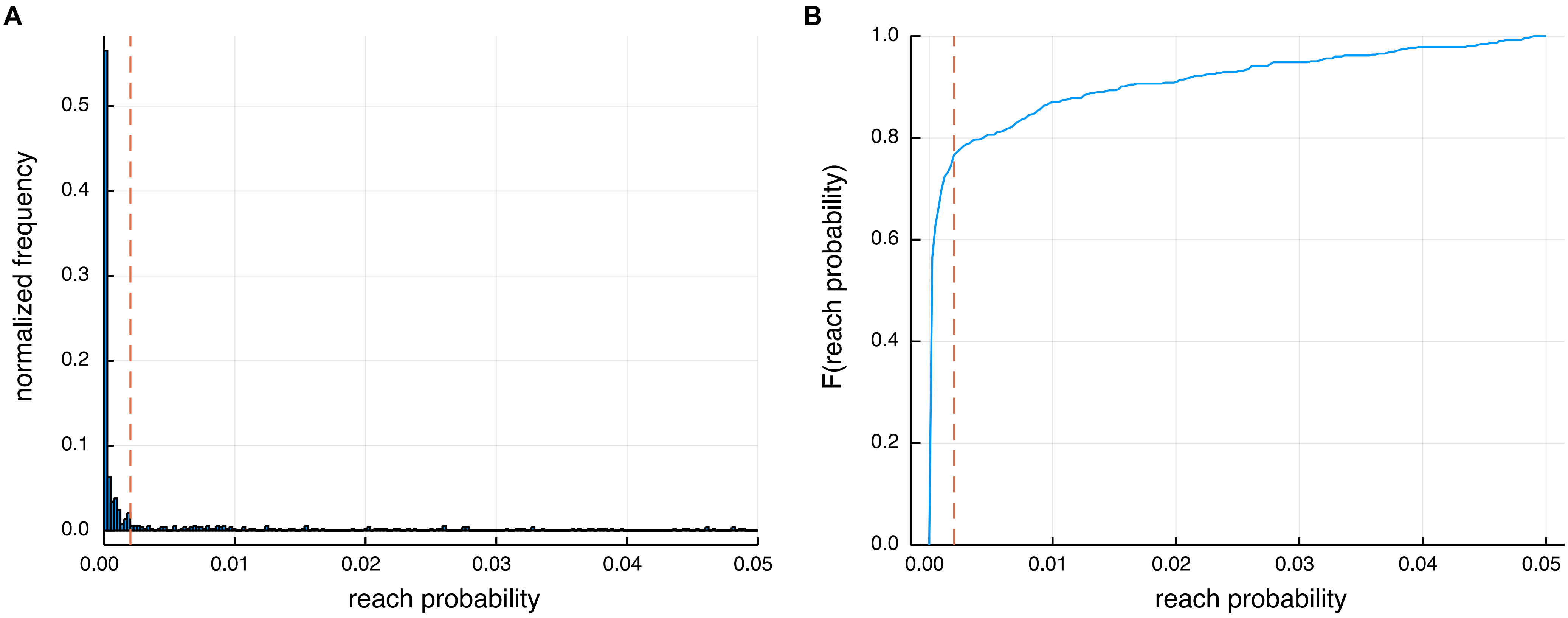
A
normalized frequency
0.5
0.4
0.3
0.2
0.1
0.0
0.00
0.01
0.02
0.03
0.04
0.05
reach probability
B
F(reach probability)
1.0
0.8
0.6
0.4
0.2
0.0
0.00
0.01
0.02
0.03
0.04
0.05
reach probability

Figure S3

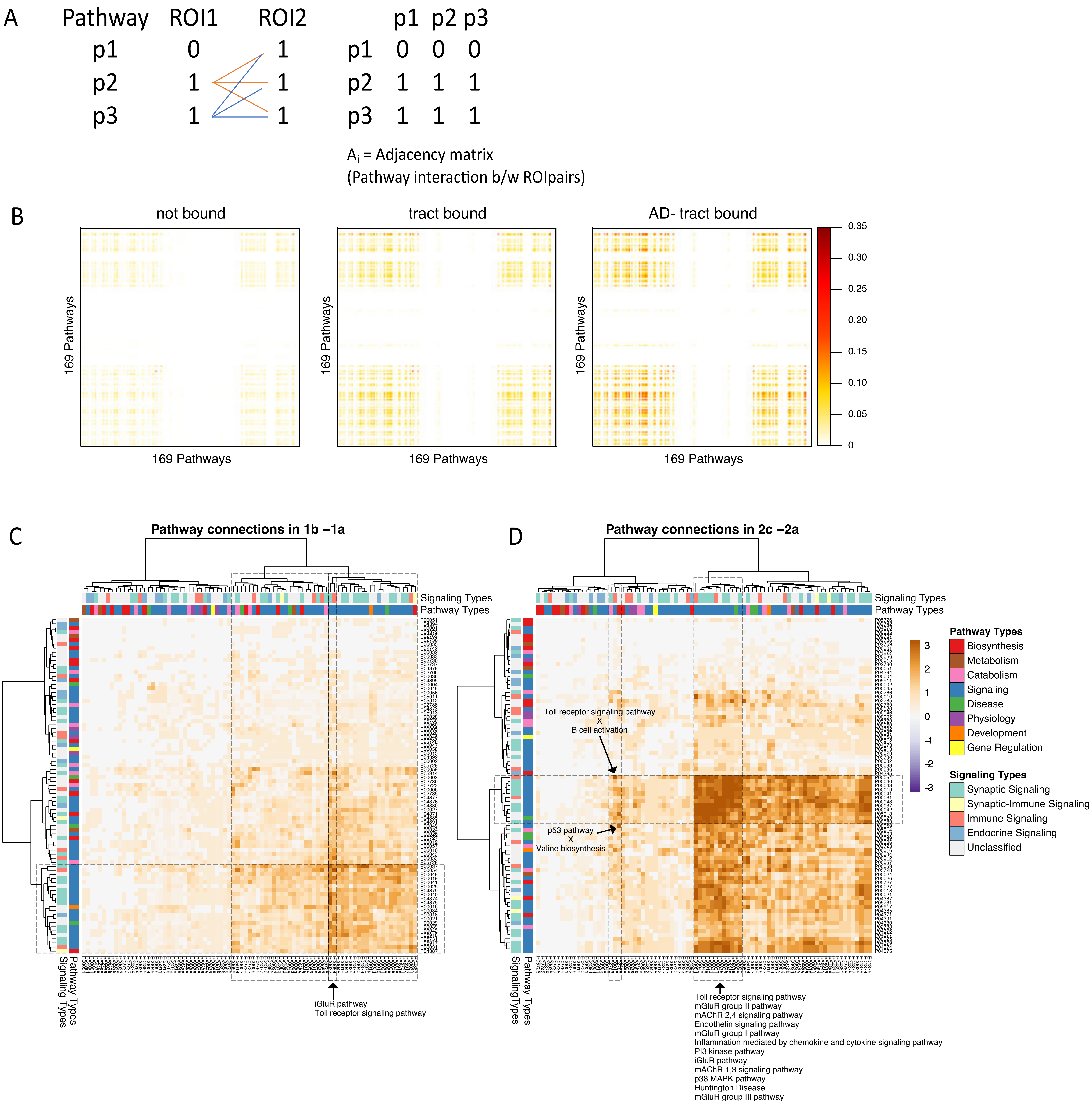
A
Pathway ROI1 ROI2
p1 0 1
p2 1 1
p3 1 1
p1 p2 p3
p1 0 0 0
p2 1 1 1
p3 1 1 1
A_i = Adjacency matrix
(Pathway interaction b/w ROIpairs)
B
not bound
tract bound
AD- tract bound
169 Pathways
0.35
0.30
0.25
0.20
0.15
0.10
0.05
0
C
Pathway connections in 1b –1a
D
Pathway connections in 2c –2a
Signaling Types
Pathway Types
Toll receptor signaling pathway
X
B cell activation
p53 pathway
X
Valine biosynthesis
iGluR pathway
Toll receptor signaling pathway
Toll receptor signaling pathway
mGluR group II pathway
mAChR 2,4 signaling pathway
Endothelin signaling pathway
mGluR group I pathway
Inflammation mediated by chemokine and cytokine signaling pathway
PI3 kinase pathway
iGluR pathway
mAChR 1,3 signaling pathway
p38 MAPK pathway
Huntington Disease
mGluR group III pathway
3
2
1
0
-1
-2
-3
Pathway Types
Biosynthesis
Metabolism
Catabolism
Signaling
Disease
Physiology
Development
Gene Regulation
Signaling Types
Synaptic Signaling
Synaptic-Immune Signaling
Immune Signaling
Endocrine Signaling
Unclassified

Figure S4

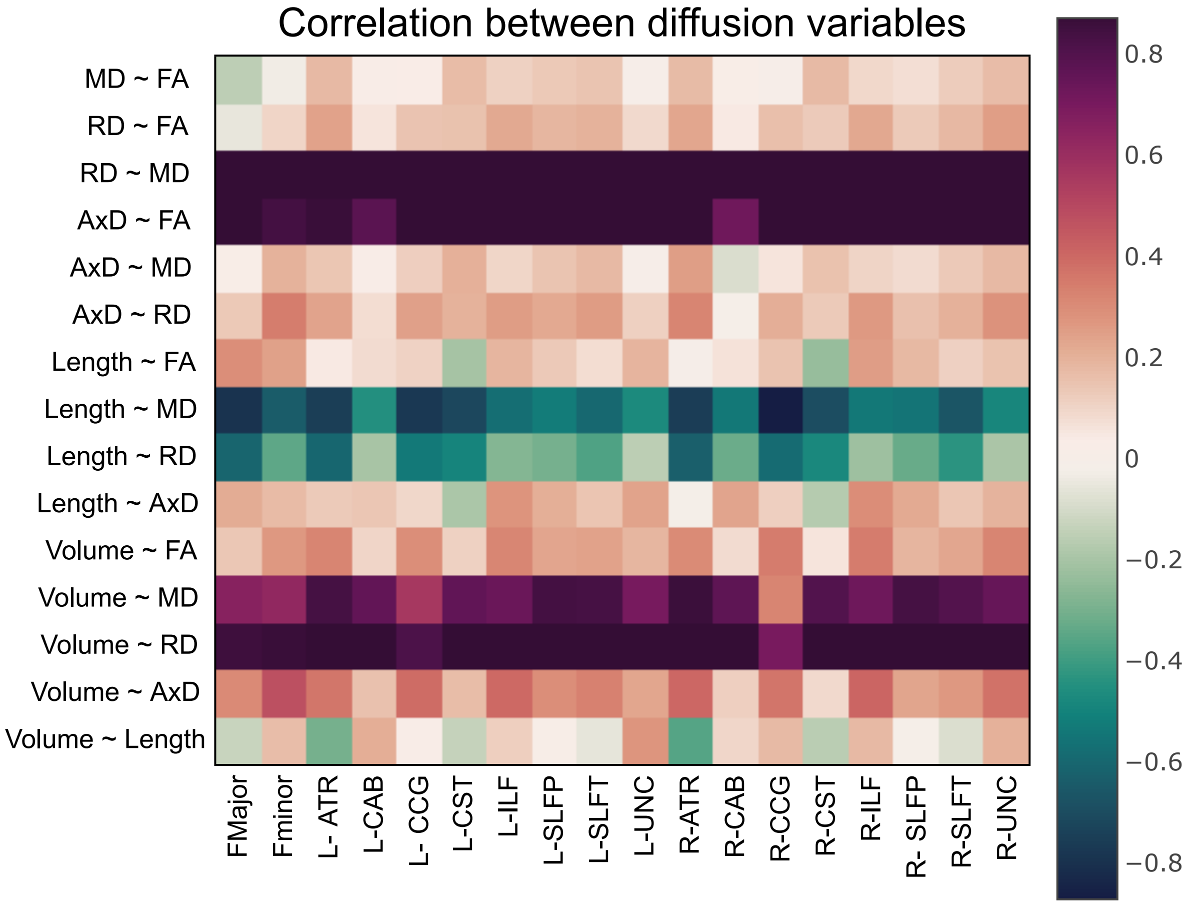
Correlation between diffusion variables
MD ~ FA
RD ~ FA
RD ~ MD
AxD ~ FA
AxD ~ MD
AxD ~ RD
Length ~ FA
Length ~ MD
Length ~ RD
Length ~ AxD
Volume ~ FA
Volume ~ MD
Volume ~ RD
Volume ~ AxD
Volume ~ Length
FMajor
Fminor
L- ATR
L-CAB
L- CCG
L-CST
L-ILF
L-SLFP
L-SLFT
L-UNC
R-ATR
R-CAB
R-CCG
R-CST
R-ILF
R- SLFP
R-SLFT
R-UNC
0.8
0.6
0.4
0.2
0
−0.2
−0.4
−0.6
−0.8
